# Evaluating a digital intervention for prevention of type 2 diabetes: protocol for a cluster-randomised controlled trial in urban and rural India

**DOI:** 10.1186/s12889-025-24337-0

**Published:** 2025-10-01

**Authors:** Parizad Avari, Harish Ranjani, Sharma Nitika, Narayanaswamy Jagannathan, Viswanathan Mohan, Ashutosh Yadav, Menka Loomba, Akansha Tyagi, Nick Oliver, Vinitaa Jha, John Campbell Chambers, Jonathan Valabhji, Ranjit Mohan Anjana

**Affiliations:** 1 School of Public Health, Imperial College London, London, UK; 2 Department of Metabolism, Digestion and Reproduction, Imperial College London, London, UK; 3 Madras Diabetes Research Foundation and Dr. Mohan’s Diabetes Specialities Centre, Chennai, Tamil Nadu India; 4 Devki Devi Foundation, New Delhi, India; 5 Lee Kong Chian School of Medicine, Nanyang Technological University, Singapore, Singapore

**Keywords:** Type 2 diabetes, Diabetes prevention, Digital intervention, mHealth, mobile app

## Abstract

**Background:**

The rising prevalence of diabetes, particularly in low- and middle-income countries, highlights an urgent need for innovative approaches to prevention. India faces a growing burden of type 2 diabetes (T2D), placing considerable strain on affected individuals and healthcare systems. This study aims to evaluate the effectiveness of a digital intervention designed to prevent T2D in individuals with pre-diabetes in India.

**Methods:**

A total of 3,240 individuals with pre-diabetes will be recruited across urban and rural settings in Tamil Nadu and Delhi. Participants will be cluster randomised to either the digital intervention group or the control group (usual standard of care). Clusters will comprise of wards in urban areas and villages in rural areas. Initial screening will be conducted using the Indian Diabetes Risk Score (IDRS) ≥ 30 and/or a random capillary blood glucose level ≥ 110 mg/dL. Final recruitment into the study will be based on capillary oral glucose tolerance testing (OGTT) and/or HbA1c levels. The primary outcome is incidence of T2D at 24 months, and secondary outcomes are cardiometabolic events, lifestyle and behavioural factors, and cost effectiveness of digital interventions. The study is registered with the Central Trials Registry of India (CTRI/2022/02/040650).

**Discussion:**

This protocol paper details the comprehensive study design aimed at evaluating the effectiveness of a digital intervention to prevent T2D in India. If effective, the study will provide important insights into the use of technology in resource-constrained settings and offer a scalable solution for diabetes prevention. The findings could also inform future interventions and policies aimed at curbing the rising burden of diabetes in low- and middle-income countries.

**Trial registration:**

CTRI/2022/02/040650 (registration date: 28.02.2022).

https://ctri.nic.in/Clinicaltrials/pmaindet2.php?EncHid=NjE2NzE=&Enc=&userName=

**Supplementary Information:**

The online version contains supplementary material available at 10.1186/s12889-025-24337-0

## Background

The rising prevalence of type 2 diabetes (T2D) and obesity represents significant public health challenges, necessitating urgent action for prevention and control. By 2045, the global prevalence of diabetes is projected to reach 700 million people [1], with cardiovascular disease (CVD) and diabetes currently contributing to 17.9 million and 1.5 million deaths annually, respectively. In India, the situation is particularly alarming, with 101 million individuals diagnosed with diabetes and 136 million with prediabetes [2].

Clinical trials have demonstrated that lifestyle interventions can prevent T2D, with benefits persisting years after the intervention ends [3, 4]. However, face-to-face programmes face challenges, including low acceptability due to time commitments, perceived stigma, and high costs, especially at scale [5]. Digital technologies offer a promising alternative, enabling personalized, lower cost and scalable interventions accessible to large populations. Digital delivery methods, such as mobile apps and text messaging, provide instant, interactive support for behaviour change, and can be tailored to individual needs.

Although most digital interventions have been studied in high-income countries [6–8], there is growing evidence of their effectiveness in low- and middle-income countries [9], including South Asia. For example, small scale studies with digital interventions in India have successfully demonstrated increased physical activity using a 100-day pedometer and an internet intervention [10]. Furthermore, a text messaging intervention with face-to-face lifestyle sessions has led to improved diet and reduced diabetes risk [11].

It is therefore timely to explore whether digital technologies can be leveraged to develop acceptable, effective, scalable and sustainable population health interventions for the prevention of T2D and CVD in South Asian men and women. In our pilot study conducted with 567 participants using a mobile app intervention, significant reductions in body weight were observed in both urban (−2.40 kg, 95% CI −3.10 to −1.69; *p* < 0.001) and rural populations (−1.19 kg, 95% CI −1.55 to −0.82; [*p* < 0.001]) [12]. The digital intervention components used in the pilot have been consolidated into a single, unified intervention for this current study.

This manuscript outlines the study protocol of the randomised controlled trial to assess the effectiveness of a digital intervention app (called My Sugar Journey) in T2D prevention compared to control (no digital intervention) among adults with prediabetes in urban and rural India.

## Methods/design

### Design

**Fig. 1:**
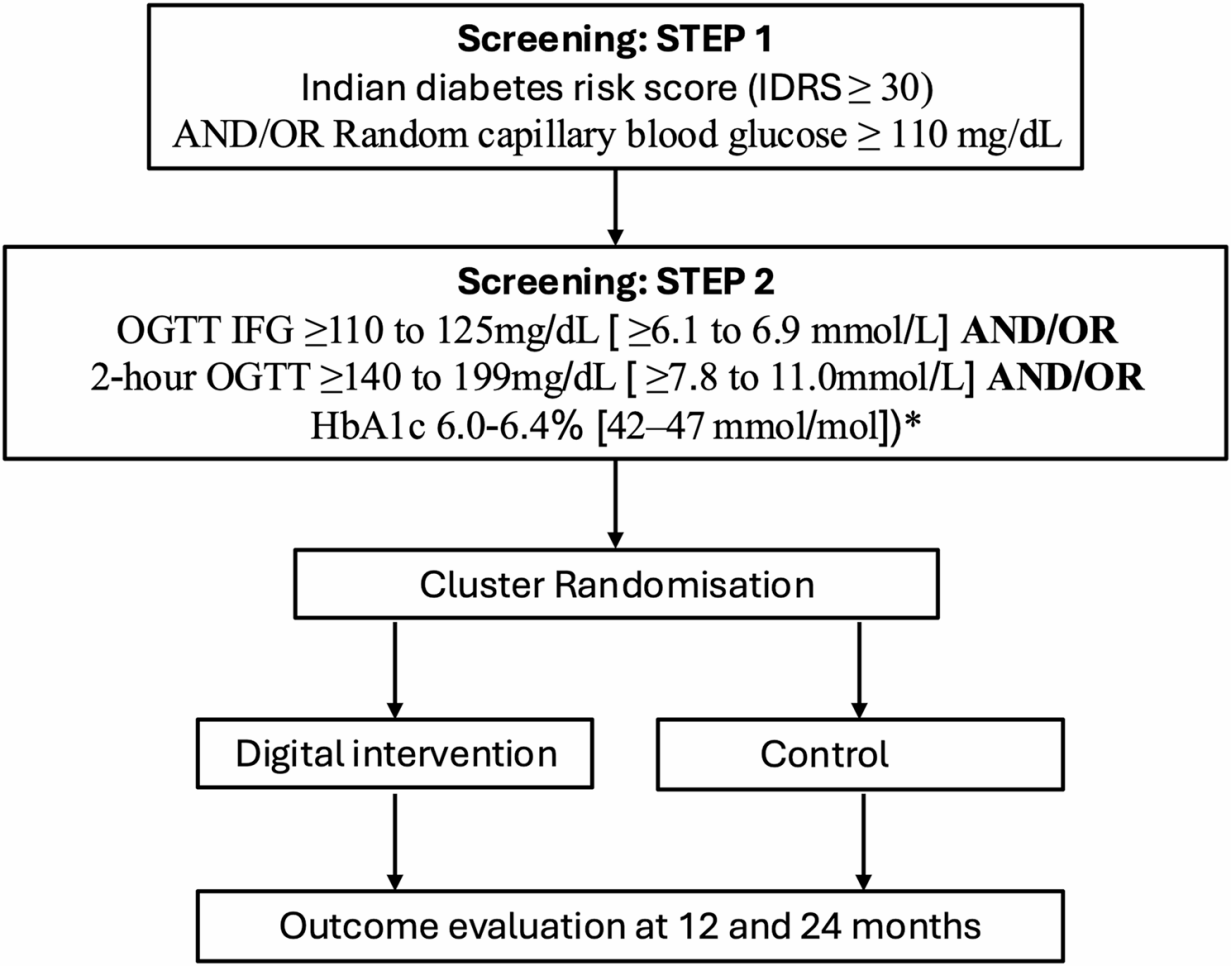
Flow diagram of the diabetes prevention intervention study. A 2-step screening phase is incorporated prior to study recruitment and randomisation. Outcome of T2D incidence will be determined by the specific criterion applied to each individual on admission into the study (i.e. fasting glucose, 2-hour glucose on OGTT, and/or HbA1c). *If haemoglobin < 10 g/L, inclusion criteria will be dependent on the fasting glucose or 2-hour post-OGTT glucose. Abbreviations: IDRS; Indian diabetes risk score; IFG, impaired fasting glucose; OGTT, oral glucose tolerance test

This is a cluster randomised, parallel group prospective study comparing the digital intervention group (Intervention group) and control group receiving usual care (Control group). (Fig. 1). A total of 3,240 participants will be recruited over 2 years from Delhi in the north (urban and rural) and Tamil Nadu (urban-Chennai and rural-Chunampet) in the south of India.

### Eligibility

#### Inclusion criteria

Participants eligible for the study must be adults aged 18 to 60 years old with an Indian Diabetes Risk Score (IDRS) of 30 or higher. IDRS is a simple, cost-effective tool developed to assess the risk of developing T2D in the Indian population and is widely used in community screening programs across India. The IDRS considers various risk factors relevant to the Indian population and assigns a score based on lifestyle, family history and anthropometric measures [13] Participants should have prediabetes, as defined by the WHO criteria [14], which includes impaired fasting glucose with levels between 110 and 125 mg/dL (6.1 to 6.9 mmol/L) and/or a 2-hour glucose level from an oral glucose tolerance test (OGTT) between 140 and 199 mg/dL (7.8 to 11.0 mmol/L) and/or an HbA1c of 6.0–6.4% (42–47 mmol/mol) based on the International Expert Committee (IEC) and National Institute for Health and Care Excellence (NICE) guidelines (rather than the definition by the American Diabetes Association (ADA)) to focus on individuals at the highest risk of conversion to diabetes [15, 16]. Participants must also be smartphone users with basic literacy skills in English, Tamil, or Hindi..

#### Exclusion criteria

Participants will be excluded from the study if they have pre-existing type 1 or type 2 diabetes, meet any criteria for type 2 diabetes (T2D) during screening, or are on oral hypoglycaemic agents or other medications that affect weight. Those who have undergone bariatric surgery, or plan to do so within the next 12 months, are also ineligible. Additionally, individuals who are pregnant, planning pregnancy, or unable to engage in physical activity as determined by their primary care provider, will not be included. Exclusion extends to those participating in other diabetes-related clinical trials, individuals with current or recent cancer (less than 12 months’ post-treatment), and anyone unable to participate due to other factors as assessed by the Chief Investigators.

### Sample size and feasibility

Based on our previous findings, the expected diabetes risk reduction based on previous face to face prevention studies has been shown to be 28–32%. We hypothesized that a digital intervention would enable a 24% reduction in the incidence of T2D (*p* < 0.05) compared to the control group. The effect size selected was based on published literature which suggests lower effectiveness for digital compared to face-face interventions.

To demonstrate that difference as significant at *p* < 0.05 and with 80% power and accounting for approximately 20% drop-out rate, 3,240 participants will be recruited.

### Ethics approval

The study has been given ethical approval for conduct in India by the Institutional Ethics Committee and registered with the Central Trials Registry of India (CTRI/2022/02/040650; registration date 28.02.2022). Each site also sought their own Institutional Ethics committee permissions from Devki Devi Foundation, New Delhi and Madras Diabetes Research Foundation, Chennai. Participant recruitment is currently ongoing.

### Participant screening

Participants will be identified through a targeted screening process conducted by the field team. During these visits, the research team will assess potential individuals based on the study’s preliminary inclusion criteria. Prior to participating in any study-specific screening activities, potential participants will be required to sign the approved informed consent form. Eligibility will be assessed through screening evaluations, and reasons for any exclusions will be recorded in a Participant Screening Log.

The study screening procedures are summarised in Fig. 1.

#### Screening STEP 1

During the first screening visit, eligibility will be evaluated based on the inclusion and exclusion criteria, the IDRS score, and random capillary blood glucose [13].

Participants meeting the criteria – defined as IDRS ≥ 30 and/or a random capillary blood glucose level ≥ 110 mg/dL – will proceed to the second stage of screening. In Asian Indians, a screening threshold of random capillary blood glucose ≥ 110 mg/dL is recommended [17].

#### Screening STEP 2

In this stage, a capillary oral glucose tolerance test (OGTT) [18, 19] will be performed and venous blood samples collected to measure HbA1c and haemoglobin levels. Additionally, anthropometric measurements, including blood pressure, height, weight, waist circumference, and body composition, will be recorded. Participants will also complete assessments related to quality of life (QOL-Eq. 5D), sleep patterns, alcohol use, smoking status, and socioeconomic status, the latter of which will be evaluated using a standardized scale relevant to India that considers occupation, income, and education – the Kuppuswamy Scale [20].

Participants meeting the inclusion criteria will be enrolled on to the study. For individuals with a haemoglobin less than 10 g/L, the HbA1c value will be disregarded, due to anaemia affecting glycated haemoglobin [21, 22]). In people with haemoglobin < 10 g/L, inclusion criteria will solely be dependent on the fasting glucose or 2-hour post-OGTT glucose. Participants diagnosed with T2D will be directed for specific diabetes care in local healthcare centres.

### Participant recruitment and cluster randomisation

**Fig. 2:**
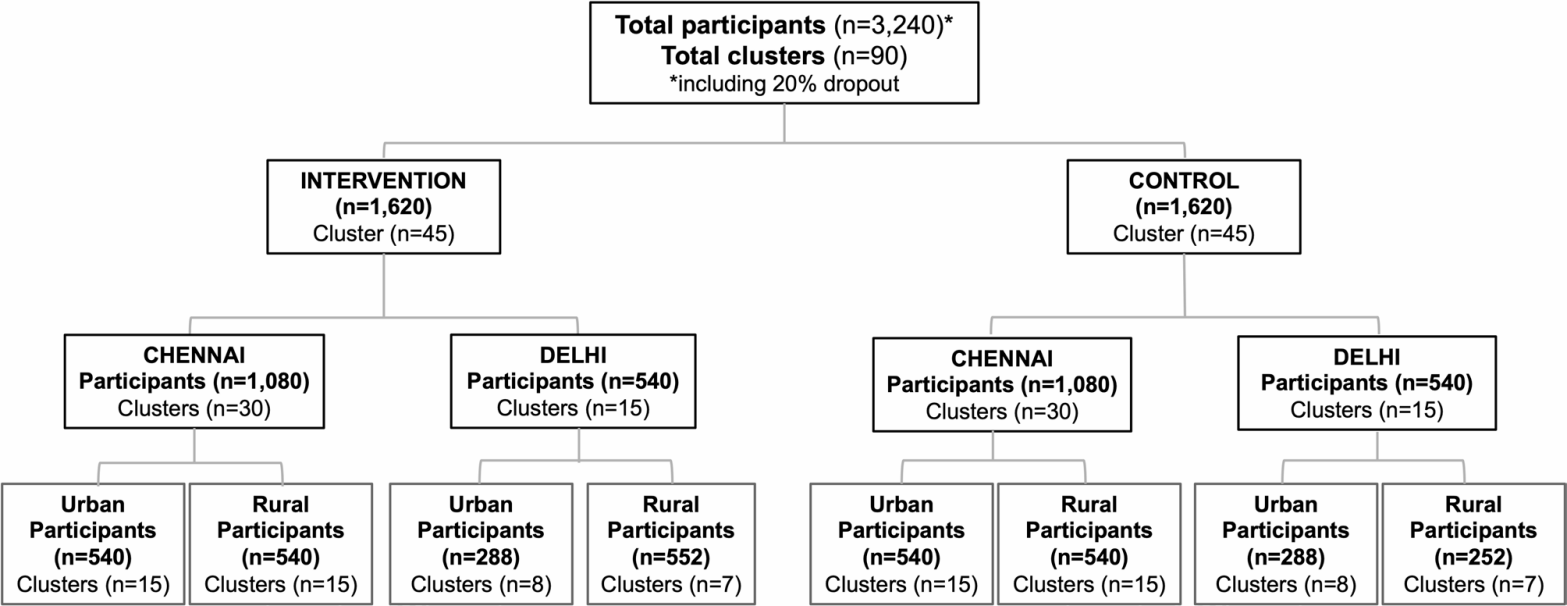
Summary of participant and cluster recruitment in urban and rural Chennai and Delhi. A total of 60 clusters (30 urban and 30 rural) will be recruited in Chennai, with 30 clusters (15 urban and 15 rural) in Delhi. Each cluster represents 36 participants to be recruited

Participants will be cluster randomised from both urban and rural areas across Tamil Nadu and Delhi (Fig. 2). Using STATA software, clusters will be randomly allocated in a 1:1 ratio to either the control group, which will receive the standard of care, or the intervention group, which will receive the digital intervention.

In Tamil Nadu, a total of 60 clusters (30 urban and 30 rural) will be established to recruit 2,160 participants (Fig. 2). The Chennai city (urban) has 200 wards, with 68 wards having a population density greater than 10,000. Each urban ward will constitute a single cluster, with 36 participants recruited per cluster. In rural areas, three villages will be grouped together to form one cluster to achieve the target of 36 participants per cluster. This approach accounts for the anticipated lower prevalence of prediabetes in rural areas compared to urban settings, requiring more extensive screening to meet the cluster size.

Similarly, in Delhi, a total of 30 clusters (15 urban and 15 rural) will be recruited. Delhi’s urban population is organized into blocks, while the rural population is divided into clusters. The study will screen 15 urban clusters (2 or 3 blocks will be combined to form a cluster) with a population density of 29,638 and 15 rural clusters with a population density of 29,434. Both in urban and rural areas, participants will be screened till the target of 36 participants per cluster is achieved.

### Study intervention

A description of activities at each visit has been outlined below and a summary provided in Table 1.

**Table 1:** Quantitative outcome measures

Outcome measures	Baseline	Month 6	Month 12	Month 24
Demographics	X			
Anthropometrics (height, body weight, waist circumference, BMI)	X	X	X	X
HbA1c	X		X	X
Capillary oral glucose tolerance test	X		X	X
Systolic and diastolic blood pressure	X	X	X	X
Food frequency questionnaire	X	X	X	X
Physical activity – MPAQ questionnaire	X	X	X	X
Lifestyle questionnaire - smoking, alcohol	X	X	X	X
Socioeconomic status tool	X			X
App engagement and usage outcomes^a^				
Total time spent per participant on app per day/week/month/entire duration	X	X		
App session interval (i.e. time between visits to the app)	X	X		
Number of sessions per participant on app per day/week/month/entire duration	X	X		
MARS questionnaire (for intervention group only)		X		
Economic outcomes				
QOL-EQ -5D	X	X	X	X
Collection of hospital care and associated costs	X	X	X	X

^a^For intervention group only

Abbreviations: *BMI*, body mass index; *HbA1c*, glycated haemoglobin; *MARS*, mobile application rating scale; *MPAQ*, Madras diabetes research Foundation - Physical activity questionnaire; *QOL-EQ-5D*, quality of life EuroQol Group-5D questionnaire

After randomization, study participants will receive instructions on how to proceed in the study according to their study group. Individuals in the control group will receive an educational session, either via telephone or will be directed to a local healthcare clinic. Standard educational materials will be provided on lifestyle risk factors of T2D and recommendations on a healthy diet and physical activity in accordance with international and national recommendations.

For participants assigned to the digital intervention group, the research team will conduct home visits to administer study questionnaires, assist with downloading the digital intervention app, and complete the onboarding process. During this visit, participants will receive a brief overview of the app’s functionality and features.

After the 6-month intervention period, participants will continue to have access to the digital app on their phones and may use it at their discretion. However, no further active engagement will be provided, such as notifications, nudges, or telephone calls. Participants are free to interact with the app’s features as they wish.

### Digital intervention development and description

**Fig. 3:**
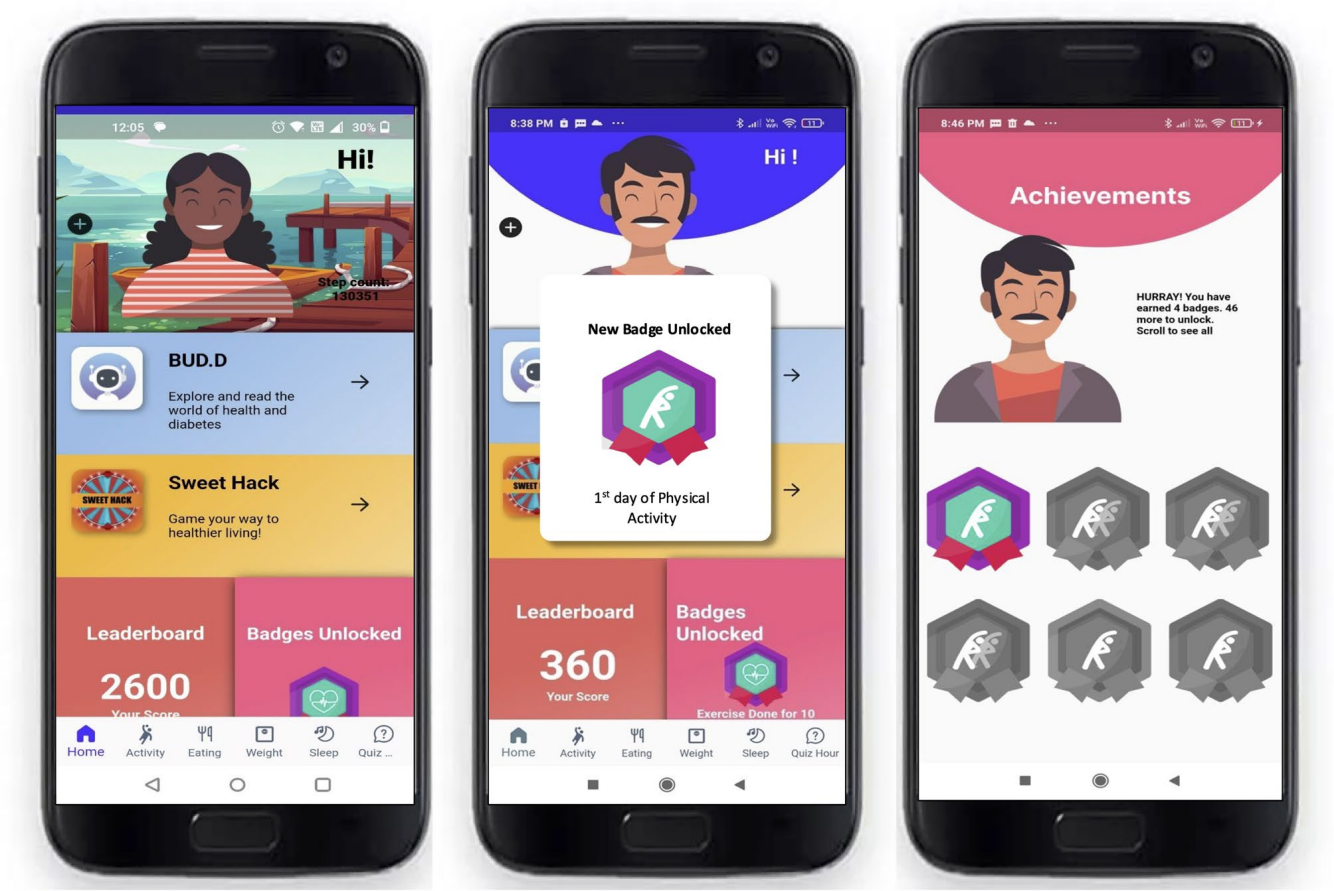
The digital intervention app, My Sugar Journey

The digital app, My Sugar Journey (Fig. 3), has been developed for use in South Asian Indian states (both in urban and rural areas) namely; from northern India Delhi and southern India Tamil Nadu (Chennai urban and Chunampet rural). Building on the lessons learned from the pilot study [12], we ensured that the app was also available in the local language commonly spoken in the two states. The development process involved careful consideration of regional factors and user preferences. The outcomes of the initial testing helped refine the app’s features for better usability and relevance.

In the beta testing phase, a total of 25 individuals from diverse backgrounds tested the app. The group included scientists, doctors, managers, diabetes educators, app developers, freelancers, illustrators and home makers, as well as some people with diabetes. This varied tester profile allowed us to gather valuable feedback from different professional perspectives, which was instrumental in fine-tuning the app for a wide range of users.

The app is available in three languages: English, Hindi and Tamil. The software is implemented as a mobile phone application and is available for download within research settings from the Google Play Store and the Apple App Store for Android and iPhone users, respectively. Once the recruited participant downloads the MSJ application, the study team sends a study code to register and use the app.

The main functionalities of the MSJ app include: (i) browsing lifestyle and behavioural suggestions, including nutrition and meal planning through recipes (ii) an artificial intelligence (AI) enabled chat-bot helping participants to navigate through various lifestyle sections (iii) gamification with each gaming level providing lifestyle and behavioural advice (iv) self-monitoring of selected behaviours (step-count, physical activity and food tracking, as well as weight tracking), (v) getting prompts and summary feedback for habit formation through push-notifications, and (vi) a brief quiz based on the content given in the app to enhance engagement.

Lifestyle goals of the intervention are to improve diet, increase physical activity, reduce sedentary behaviour, reduce excess body weight, improve sleep, reduce stress, cease (or at least decrease) smoking, and moderate alcohol consumption. The targets for diet and lifestyle are based on the American Diabetes Association recommendations [23].

Participants enrolled in the digital intervention arm receive once monthly telephone calls for the 6 months intervention (a maximum of 6 telephone calls in total), only if there is no engagement with the app over any particular month. App engagement will be monitored based on app usage from the backend data team. During the telephone call, participants are simply asked if any technical issues and gently prompted to use the app. The anticipated duration of the telephone call is approximately 1–2 min. From our pilot data conducted during the Covid-19 period [12], there was a relatively high dropout rate, and hence engagement telephone calls have been incorporated. Research personnel making telephone calls do not need specific training in diabetes or dietetics, as the objective is to serve as a reminder to participants to engage with the app, keeping the support cost-effective and accessible.

### Primary and secondary outcomes

The primary outcome is incidence of T2D at 24 months (as determined by the specific criterion applied to each individual on their admission into the study (fasting glucose, 2-hour glucose on OGTT, and/or HbA1c). For example, if an individual’s prediabetes status was defined by HbA1c value (within the range 42-47mmol/mol), their progression to type 2 diabetes will be defined by the associated HbA1c threshold (48mmol/mol or greater). Changes over 6 and 12 months are considered secondary outcomes.

Secondary outcomes are listed in Table 1 and include changes in cardiometabolic outcomes; intervention uptake, use and satisfaction; behavioural and clinical outcomes; and costs related to implementation. Data will be collected at baseline, and 6, 12 and 24 months as outlined in Table 1.

### Qualitative outcomes

Between 6 and 12 months into the study, focus group discussions will be conducted with up to 15 participants from both urban and rural areas of Chennai and Delhi. Interviewees will be selected through purposive sampling, aiming for variation across geographical area, socioeconomic background and areas of high and low uptake. The aim is to explore the challenges and barriers associated with using digital interventions for lifestyle behaviour modification among individuals living in South Asia. Semi-structured interviews will be conducted using pre-designed guides by trained interviewers.

### Statistics and data analysis

The primary analyses will be based on an intention to treat (ITT) principle. All participants who are randomised into the study will be included in the ITT analysis and the analysis is conducted according to the randomised treatment arm. Outcomes will be assessed for normal distribution.

For the primary outcome, the incidence of T2D in each study arm will be determined based on the participants’ entry criterion. Change in HbA1c, fasting glucose, or 2-hour OGTT glucose values will be calculated from baseline to endpoint (at 12 months and 24months) and reported based on the specific criterion applied to each individual on their admission into the study. Outcomes will be assessed by two-tailed unpaired t-test if normally distributed or the Wilcoxon Rank sum test if non-normally distributed.

## Discussion

This study is the largest study to assess the effectiveness of a digital app intervention for the prevention of T2D in India to date. The adoption of digital tools for health behaviour modification offers a scalable and potentially cost-effective approach, particularly in settings with limited healthcare resources. However, realising their full potential, requires addressing several challenges.

A key challenge is the variation in digital literacy and varying levels of access to smartphone technology, particularly in rural populations. Rural India has experienced rapid internet adoption, driven by the surge in online services such as digital payments, e-learning, and telemedicine during the COVID-19 pandemic. With more than 821 million users, rural India has more internet users than urban India [24]. The Indian government’s Digital India Program has also played a crucial role in not only promoting digital literacy, but also providing infrastructure in rural areas [25]. Despite this, disparity in internet access across India poses another significant barrier, and although internet penetration is increasing, many rural areas still experience inconsistent connectivity. To mitigate this, low-data usage was incorporated into the design of the app. Furthermore, the app is designed to accommodate diversity, offering content in three languages with dietary and lifestyle information tailored to the local population. Language-agnostic features, such as gaming elements, further enhance accessibility, making the app more inclusive for users with varying literacy levels.

Identifying and reaching individuals at high risk can be challenging, particularly in rural areas [26]. The protocol includes proactive strategies to engage these populations effectively. Cluster randomisation was employed in rural areas to prevent cross-contamination between groups, as close-knit communities may increase the risk of intervention overlap [27].

Another key factor in determining the success of the intervention is user adherence and engagement with the app. The study intervention offers a personalised approach to enhance individual engagement through gamification and motivation strategies, such as challenges, rewards, and progress tracking. By offering a more interactive experience, the app encourages sustained participation and adherence to healthy lifestyle changes, which are necessary for T2D prevention [23]. App data usage will be used to assess uptake with the digital intervention.

Finally, due to the high prevalence of anaemia in the South Asian population, which can affect HbA1c readings and lead to inaccurate assessments of diabetes risk [21, 22], recruitment is not based solely on HbA1c levels. This dual approach ensures more accurate identification of high-risk individuals. Another challenge in conducting studies in rural settings is the difficulty of using venous sampling and transporting blood samples to centralised laboratories, particularly due to limited infrastructure, logistical constraints and time taken. To address this, the study utilises capillary OGTT for more practical and immediate results in the field [28].

In conclusion, despite the challenges, the use of a digital platform reduces the need for extensive physical infrastructure and human resources, offering a scalable solution that could be highly cost-effective in resource-constrained environments. If proven successful, the study has the potential to contribute to the growing body of evidence supporting digital health solutions for diabetes prevention, with broader implications for public health policy and healthcare delivery in resource-constrained settings.

## Supplementary Information

Supplementary material 1. 

## Data Availability

No datasets were generated or analysed during the current study.
